# Comparison of six COVID-19 serology assays for detection of antibodies from patients infected with ancestral and a spectrum of SARS-CoV-2 variants

**DOI:** 10.1099/acmi.0.000974.v3

**Published:** 2025-07-07

**Authors:** Rachel Lau, Chandrika Senthilkumaran, Jeffrey Chong, Freda Qi, Rosmol-Stanes Pulikkottil, Jennifer Ma, Katherene Ogbulafor, Larry Gabe, Kathy Manguiat, Alyssia Robinson, Heidi Wood, Angel Xinliu Li, Mohammad Mozafarihashjin, Aaron Campigotto, Allison J. McGeer, Samira Mubareka, Jonathan B. Gubbay, Julianne V. Kus, Shelly Bolotin, Melissa Richard-Greenblatt, Vanessa Tran

**Affiliations:** 1 Public Health Ontario, Toronto, Ontario, Canada; 2 Public Health Agency Canada, Ottawa, Ontario, Canada; 3 Lunenfeld-Tanenbaum Research Institute at Mount Sinai Hospital, Sinai Health, Toronto, Ontario, Canada; 4 National Microbiology Laboratory, Public Health Agency of Canada, Winnipeg, Manitoba, Canada; 5 Department of Microbiology, Sinai Health System, Toronto, Ontario, Canada; 6 Department of Internal Medicine, Rochester General Hospital, Rochester, NY, USA; 7 Department of Microbiology, The Hospital for Sick Children, Toronto, Ontario, Canada; 8 Department of Laboratory Medicine and Pathobiology, University of Toronto, Toronto, Ontario, Canada; 9 Dalla Lana School of Public Health, University of Toronto, Toronto, Ontario, Canada; 10 Department of Microbiology, Sunnybrook Health Sciences Centre, Toronto, Ontario, Canada; 11 BC Children’s Hospital, Vancouver, British Columbia, Canada

**Keywords:** coronavirus disease 2019 (COVID-19), plaque reduction neutralization test, serology, severe acute respiratory syndrome coronavirus 2 (SARS-CoV-2), variants of concern

## Abstract

Serology assays against spike, receptor binding domain (RBD) and nucleocapsid proteins of the severe acute respiratory syndrome coronavirus 2 are essential for serosurveillance. We performed a comparison of four medium-to-high throughput commercial assays [Abbott Laboratories, Ortho Clinical Diagnostics, Meso Scale Diagnostics (MSD)], one point-of-care test (ZEKMED) and a laboratory-developed plaque reduction neutralization test using a reference panel and clinical specimens. Overall, the assays showed a high positive percent agreement of ≥85% and negative percent agreement of ≥90%, with the MSD anti-spike IgG assay having the best performance (100% in both). Notably, Abbott anti-nucleocapsid IgG, MSD anti-spike IgG and ZEKMED anti-spike RBD IgM/IgG combined assays were able to detect antibodies from individuals infected with all different variants tested – Alpha, Beta, Gamma, Delta and Omicron. The limit of detection (LOD) of the assays ranged from 9.9 to 62.0 BAU ml^−1^, with the Abbott anti-spike RBD having the lowest LOD. The COVID-19 serology assays will continue to be useful in determining seroprevalence from infection and vaccination.

## Data Summary

All available data are presented in this manuscript. In brief, 95% CI were calculated with MedCalc diagnostic test evaluation calculator [1]. ANOVA and Tukey HSD to compare values from quantitative assays to PRNT_50_ were performed by R version 4.3.1 in R Studio 2023.9.1 Build 494.

## Introduction

Coronavirus disease 2019 (COVID-19) caused by the severe acute respiratory syndrome coronavirus 2 (SARS-CoV-2) has had a global impact with a cumulative total of 776 million infections and 13.6 billion doses of vaccine administered worldwide as of October 2024 [2]. Variants of concern (VOCs) are genetic variants of SARS-CoV-2 as defined by WHO with increased transmissibility and increased virulence in clinical disease or have an impact on health systems, of which some may have the ability to thwart public health measures including diagnostics, vaccines and therapeutics [3]. Since the pandemic, five VOCs (Alpha, Beta, Gamma, Delta and Omicron) have been assigned and circulated globally [4]. After the emergence of Omicron, although not designated as VOCs, SARS-CoV-2 continues to evolve, giving rise to new lineages, including BA.1, BA.5 and XBB.1 to the more recent JN.1, XEC and LP.8.1 [5].

COVID-19 diagnostic tools, including PCR and rapid antigen tests, are the mainstay for the detection of acute infections [6,8]. In contrast, serological assays offer limited diagnostic utility because of the low sensitivity in the acute phase of infection when individuals are most infectious but remain valuable for epidemiological investigations and serosurveillance [9]. Generally, SARS-CoV-2 antibody levels become detectable 2 weeks post-symptom onset (PSO) and remain detectable for at least 6 months following infection [10,11]. Some mild and asymptomatic cases do not develop a measurable level of antibodies [12]. In some countries, including Canada, where spike mRNA or antigen COVID-19 vaccines are used, serosurveillance testing using both anti-spike (S) and anti-nucleocapsid (N) assays has attempted to estimate COVID-19 seroprevalence from vaccination versus infection [13,15]. It is important to note that the antigens cannot specifically distinguish antibodies from SARS-CoV-2 vaccination or infection; however, inferences can be made when interpreting both anti-S and anti-N together and with knowledge of the type of vaccine used in the population.

Many COVID-19 serology assays have been developed, including medium-to-high throughput commercial tests, rapid point-of-care tests (POCT) and laboratory-developed plaque reduction neutralization tests (PRNTs) that specifically detect neutralizing antibodies. The majority of these assays use antigens from the nucleocapsid, spike or spike receptor binding domain (RBD) of SARS-CoV-2; however, the antibody isotype may vary (e.g. IgM, IgG or both). IgM antibodies are generated earlier in the infection at around 11–15 days PSO but start to decline around 30 days, whereas IgG peaks at 16–30 days PSO and circulates for ~6 months [16,18].

Most commercial assays detect both binding and neutralizing antibodies but are unable to differentiate between them. PRNT is considered the ‘gold standard’ for detecting neutralizing antibodies and therefore is often used as the reference method in comparison studies [19]. The choice of serology test will depend on the intended use; however, information on how different types of assays (i.e. qualitative, quantitative, POCT, neutralization) directly compare with each other is limited, particularly in the context of different variants. These assays often have their own arbitrary units and different cut-offs for detectable antibodies, which provides an additional challenge to directly compare performance and limit of detection (LOD). A number of studies have compared performance characteristics of serology assays with variable results; however, to the best of our knowledge, studies that have assessed their abilities to detect antibodies generated from infection with VOCs are limited [20,26].

With the continual emergence of new SARS-CoV-2 lineages and updates of strains in vaccines, it is important to identify immunology assays that can detect antibodies generated from infections and vaccines of different variants. The objective of this study was to directly compare the performance of six COVID-19 serology assays, including four medium-to-high volume qualitative and quantitative commercial assays, a POCT and our laboratory-developed PRNT, using a reference panel of specimens from Canada’s National Microbiology Laboratory (NML) and the WHO International Standard for anti-SARS-CoV-2 immunoglobulin. Notably, unlike previous studies, we also include convalescent sera from VOC-infected patients. Findings from this study can provide guidance to laboratories for the selection of appropriate COVID-19 serological assays for their setting and intended use.

## Methods

### Commercial assays

A summary of the serology assays and their respective targets is listed in Table 1. We evaluated three automated high-throughput COVID-19 chemiluminescent microparticle immunoassays (CMIAs)/chemiluminescent immunoassay (CLIA) – two semi-quantitative and one quantitative: SARS-CoV-2 IgG on the Architect and Alinity i (Reference # 06R9022, Abbott Laboratories) cut-off at signal-to-cut-off ratio (S/Co) 1.4, VITROS anti-SARS-CoV-2 IgG on the VITROS 3600 (Reference # 6199919, Ortho Clinical Diagnostics) cut-off at S/Co 1.0 and SARS-CoV-2 IgG II Quant on the Alinity i (Reference # 06S6122, Abbott Laboratories) cut-off at 50 arbitrary units ml^−1^ (AU ml^−1^). All assays were performed following manufacturer’s instructions. We followed the manufacturer’s instructions for interpretation of results such that values equal to or greater than the respective cut-offs were considered positive and specimens with values less than the cut-offs were considered negative.

**Table 1.** Serology assays evaluated in this study

Manufacturer	Assay name	Detection	Assay type	Cut-off value
Abbott Diagnostics	SARS-CoV-2 IgG	Anti-N IgG	CMIA (semi-quantitative, high throughput)	1.4 S/Co
Abbott Diagnostics	SARS-CoV-2 IgG II Quant	Anti-S RBD IgG	CMIA (quantitative, high throughput)	50 AU ml^−1^
Ortho Diagnostics	VITROS anti-SARS-CoV-2 IgG	Anti-S IgG	CLIA (semi-quantitative, high throughput)	1.0 S/Co
MSD	V-Plex^®^ Coronavirus Panel 2 IgG,V-Plex^®^ SARS-CoV-2 Panel 2 IgG	Anti-N IgG,Anti-S IgG,Anti-S1 RBD IgG	Multiplex ELISA (quantitative, medium throughput)	Anti-N, 5,000 AU ml^−1^Anti-S, 1,960 AU ml^−1^Anti-S1 RBD, 538 AU ml^−1^
ZEKMED	COVID-19 IgG/IgM Rapid Test Cassette	Anti-S RBD	Lateral flow POCT (qualitative)	Qualitative
In-house	PRNT	Anti-SARS-CoV-2 (Pango lineage B.1.2)	Neutralization (quantitative, low throughput)	1:20 dilution

AU, arbitrary unit; CLIA, chemiluminescent immunoassay; CMIA, chemiluminescent microparticle immunoassay; ELISA, enzyme-linked immunosorbent assay; POCT, point-of-care test; PRNT, plaque reduction neutralization test; RBD, receptor binding domain; S/Co, signal-to-cut-off ratio.

In addition, we evaluated a commercial quantitative multiplex COVID-19 ELISA, which simultaneously detects three antibodies: anti-N, anti-S and anti-S1 RBD IgG antibodies [V-Plex^®^ Coronavirus Panel 2 or SARS-CoV-2 Panel 2 on QuickPlex SQ 120 MM platform; Reference # K15369U, K15383U, Meso Scale Diagnostics (MSD)]. Briefly, 10 µl of sample was diluted to 1:10,000 with diluent-100 provided by the manufacturer, and ELISA was performed according to kit instructions and interpreted using the manufacturer-provided cut-offs (anti-N IgG was ≥5,000, anti-S IgG was ≥1,960 and anti-S1 RBD IgG was ≥538 AU ml^−1^). Samples with results outside the instrument detection range were re-run at a lower and higher dilution if it was below and above the detection range, respectively. A conversion ratio to convert arbitrary concentration (AU ml^−1^) to the standardized WHO binding antibody unit (BAU ml^−1^) was provided by MSD.

The COVID-19 IgG/IgM Rapid Test Cassette (ZEKMED) is a point-of-care lateral flow chromatographic immunoassay that provides separate detection of anti-S RBD IgM and IgG. Briefly, 10 µl of neat sample was loaded onto the cassette, followed by two drops of buffer. After 15–20 min, results were read by two blinded independent readers. Discordant results between the two readers were considered ‘equivocal’.

### Plaque reduction neutralization test

SARS-CoV-2 PRNT was performed as previously described [27,28]. In brief, 50 µl of serum was diluted 1:10 in Dulbecco’s Modified Eagle Medium (DMEM) supplemented with 2% FBS and 1% penicillin–streptomycin and heat-inactivated at 56 °C for 30 min. Samples were then serially diluted two-fold up to 1:320. An equal volume of SARS-CoV-2 challenge virus (Pango lineage B.1.2 that is genetically close to the ancestral strain) was then added to give a final serum dilution from 1:20 to 1:640. The virus–serum mix was then incubated at 37 °C with 5% CO_2_ for 1 h for neutralization. After incubation, the mixture was added to 12-well plates containing Vero E6 cells (ATCC^®^ CRL-1586^™^) in duplicates with a concentration of 2.0×10^5^ cells well^−1^. The final concentration of virus was 50 Plaque-forming units (PFU) well^−1^. Back titration plates containing 50, 25, 5 and 0 PFU well^−1^ were used as control plates. Specimens and control plates were incubated at 37 °C with 5% CO_2_ for 1 h with rocking every 20 min. After, 1.5 ml of 1.5% CM-cellulose was added to each well and plates were incubated at 37 °C with 5% CO_2_ for 72±2 h. Following 72 h of incubation, the overlay was removed, and cells were fixed with 10% neutral-buffered formalin for at least 1 h. Cells were stained with 0.5% crystal violet for 10 min, washed with water and air-dried. Plaques were counted and compared with the back titration controls. The highest dilution resulting in a 50% reduction in plaques compared with the controls was defined as the PRNT_50_. If there was no 50% reduction in plaques at the lowest dilution (1:20), it was considered negative for neutralization.

### Specimens and performance testing

Specimens used in this study are summarized in Table 2. Accuracy [positive percent agreement (PPA) and negative percent agreement (NPA)] was evaluated with a reference panel provided by the NML (Winnipeg, Canada). This reference panel consisted of 60 pre-VOC (collection dates from 13 May 2020 to 22 August 2020) SARS-CoV-2 antibody positive plasma from unvaccinated COVID-19 patients that were confirmed to be positive by real-time PCR and tested by PRNT and 21 pre-COVID-19 negative plasma and serum specimens (collected prior to December 2019) [29]. The 60 positive specimens were collected from Canadian Blood Services (CBS) locations in the provinces of Ontario and British Columbia. Negative specimens were collected from CBS in British Columbia and Cadham Provincial Laboratory in the province of Manitoba. PPA (comparator test positive/reference method positive) and NPA (comparator test negative/reference method negative) were determined using reference results (PRNT_50_) provided by the NML. In brief, 95% CI were calculated with the MedCalc diagnostic test evaluation calculator [1]. ANOVA and Tukey HSD to compare values from quantitative assays to PRNT_50_ were performed by R version 4.3.1 in R Studio 2023.9.1 Build 494.

**Table 2.** Sample characteristics

Specimen	Analysis	No.
Pre-VOC and pre-COVID	Accuracy	81 (60 positives, 21 negatives)
Alpha B.1.1.7 (Convalescent)	VOC antibody detection	5
Beta B.1.351 (Convalescent)	VOC antibody detection	1
Gamma P.1 (Convalescent)	VOC antibody detection	3
Delta B.1.617 (Convalescent)	VOC antibody detection	3
Omicron BA.1; BA.1.1 (Convalescent, vaccinated individuals)	VOC antibody detection	3
WHO International Standard	LOD	Panel of 8, 10-fold dilutions
Serum or plasma positive for HBV, HCV, HIV, CMV and MMR antibodiesPlasma from patient infected with parainfluenza virus	Cross-reactivity	1 each of HBV, HCV, HIV, CMV, MMR and parainfluenza, total of 6

To evaluate if mutations in VOCs impact the performance of COVID-19 serology assays, we tested specimens from VOC-infected patients. Convalescent sera from VOC-infected patients were provided by the Toronto Invasive Bacterial Diseases Network (Toronto, Canada). Convalescent sera (collected >21 days from symptom onset) were collected from unvaccinated patients infected with Alpha (*n*=5), Beta (*n*=1), Delta (*n*=3) and Gamma (*n*=3) VOCs. With the vaccine roll-out in Ontario, convalescent sera were collected from Omicron-infected patients with lineages BA.1 and BA.1.1 (*n*=3) who were also vaccinated with spike-based mRNA or adenovirus ancestral strain vaccine.

LOD was evaluated with the WHO international standard for anti-SARS-CoV-2 immunoglobulin NIBSC code: 20/136 (Cedarlane, Burlington, Canada). The standard was reconstituted with water to a concentration of 1,000 BAU ml^−1^ and serially diluted in 10-fold dilutions to 0.01 BAU ml^−1^. Each titration was run in duplicates. For MSD, the WHO standard at each concentration was further diluted 1:1,000 prior to running the assay to be within detection range. The result was plotted against each WHO standard concentration, and a linear regression was calculated to estimate the LOD of each assay in WHO units (BAU ml^−1^).

Cross-reactivity was evaluated with plasma or sera specimens from Public Health Ontario’s laboratory that have tested positive for antibodies reactive to hepatitis B virus (HBV), hepatitis C virus (HCV), human immunodeficiency virus (HIV), cytomegalovirus (CMV) and measles virus, mumps virus and rubella virus (MMR) (Table 2). In addition, sera from a patient with PCR-confirmed parainfluenza virus infection (collected 36 days from the PCR test) were included for investigation of cross-reactivity to antibodies to a respiratory virus.

Precision (intra-assay repeatability and inter-assay reproducibility) was determined with positive and negative clinical specimens. In situations where there was insufficient volume in the clinical specimens to complete precision testing, the manufacturer’s kit-provided positive and negative controls were used. Repeatability was determined with 3–20 technical replicates performed in the same run and reproducibility by 1–4 replicates performed on 4–5 separate days.

For assays with quantitative and semi-quantitative results, the coefficient of variation (%CV) was calculated using the positive samples. A %CV ≤10 was deemed acceptable.

## Results

### Performance characteristics

Overall, the enzyme immunoassays (EIAs) (Abbott, Ortho, MSD) that use spike or RBD targets had PPAs and NPAs ranging from 95 to 100% (Table 3). Abbott Quant, MSD anti-S IgG and MSD anti-S1 RBD IgG had 100% PPA, whereas the Ortho had a slightly lower PPA of 95%. Ortho and MSD anti-S IgG had 100% NPA, while Abbott Quant and MSD anti-S1 RBD IgG had lower NPAs of 95.2 and 90.5%, respectively.

**Table 3.** Performance characteristics

Assay	Detection	PPA(%) [95% CI], (*n*)	NPA(%) [95% CI], (*n*)
**Abbott**	Anti-N IgG	85.0 [73.4 to 92.9],(51/60)*	100 [83.9 to 100],(21/21)*
**Abbott Quant**	Anti-S RBD IgG	100 [94.0 to 100],(60/60)	95.2 [76.2 to 99.9],(20/21)
**Ortho**	Anti-S IgG	95.0 [86.1 to 99.0],(57/60)*	100 [83.9 to 100],(21/21)*
**MSD**	Anti-N IgG	96.7 [88.5 to 99.6],(58/60)	95.2 [76.2 to 99.9],(20/21)
	Anti-S IgG	100 [94.0 to 100],(60/60)	100 [83.9 to 100],(21/21)
	Anti-S1 RBD IgG	100 [94.0 to 100],(60/60)	90.5 [69.6 to 98.8],(19/21)
	Combined	100 [94.0 to 100],(60/60)	85.7 [63.7 to 97.0],(18/21)
**ZEKMED**	Anti-S RBD IgM†	53.3 [40.0 to 66.3],(32/60)	100 [83.9 to 100],(21/21)
	Anti-S RBD IgM‡	43.3 [30.6 to 56.8],(26/60)	100 [83.9 to 100],(21/21)
	Anti-S RBD IgG†	85.0 [73.4 to 92.9],(51/60)	100 [83.9 to 100],(21/21)
	Anti-S RBD IgG‡	80.0 [67.7 to 89.2],(48/60)	100 [83.9 to 100],(21/21)
	Combined†	93.3 [83.8 to 98.2],(56/60)	100 [83.9 to 100],(21/21)
	Combined‡	91.7 [81.6 to 97.2],(55/60)	100 [83.9 to 100],(21/21)
**PRNT_50_**	Anti-SARS-CoV-2 (Pango lineage B.1.2)	90.0 [79.5 to 96.2],(54/60)	100 [83.9 to 100],(21/21)

*Results previously published [29].

†Equivocal results considered as positives.

‡Equivocal results considered as negatives.

The MSD and Abbott assays that detect IgG antibodies to nucleocapsid proteins had PPAs of 96.7 and 85% and NPAs of 95.2 and 100%, respectively. Compared to assays that detect IgG antibodies to spike and RBD proteins, nucleocapsid assays had lower PPAs. When combining all the targets (spike, S1 RBD and nucleocapsid) in the MSD assay, PPA increased to 100%, but NPA decreased to 85.7%. ZEKMED POCT also had a lower PPA for both IgG (80–85%) and IgM (43.3–53.3%). When combining the positivity of IgG and IgM, PPA increased to 91.7–93.3% (Table 3). NPA was 100% for both IgG and IgM. Our in-house PRNT assay had 90% PPA and 100% NPA.

Linear regression of log Abbott Quant RBD values demonstrated a good correlation with log MSD spike (*R*^2^=0.8532) and S1 RBD (*R*^2^=0.8758). As expected, a weak correlation was established between Abbott Quant RBD and MSD nucleocapsid values (*R*^2^=0.3151). Specimens with higher PRNT_50_ titres, which are proportional to higher concentrations of neutralizing antibodies, were associated with higher MSD anti-S (*P*=0.0002), MSD anti-S1 RBD (*P*=0.0010) and Abbott Quant RBD (*P*<0.0001) values. PRNT_50_ titres were not proportional to MSD anti-nucleocapsid values (*P*=0.3263).

### Detection of VOC antibodies

Fifteen specimens from patients infected with various VOCs [Alpha (*n*=5), Beta (*n*=1), Delta (*n*=3), Gamma (*n*=3) and Omicron (*n*=3)] were used to test for reactivity of the assays for antibodies to different variants. Abbott anti-N IgG (15/15) and combined ZEKMED anti-S RBD IgM/IgG assays (13/13, only one of three Omicron was evaluated) were able to detect antibodies from all variants tested (Table 4). Abbott Quant anti-S RBD IgG detected antibodies from Alpha (5/5), Delta (3/3) and Gamma (2/2) but failed to detect antibodies from Beta (0/1). Ortho anti-S IgG also failed to detect antibodies from Beta but detected antibodies from Alpha (5/5), Delta (3/3), Gamma (3/3) and Omicron (3/3). MSD anti-S IgG detected antibodies from all variants (15/15); anti-S1 RBD IgG detected all variants except Beta (14/15); anti-N IgG was able to detect antibodies from Alpha (5/5), Beta (1/1), Delta (3/3), Gamma (3/3) and one of the three Omicron specimens (1/3). Our in-house PRNT detected antibodies from Alpha (5/5), Delta (3/3) and Omicron (3/3) but detected two of the three Gamma (2/3) and failed to detect Beta. Abbott anti-N IgG, MSD anti-S IgG and the combined ZEKMED anti-S RBD IgM/IgG were able to detect antibodies from all variants, while other assays detected the majority of the variants.

**Table 4.** Percent positivity (*n*/*N*) of sera from VOC-infected patients

Assay	Detection	Alpha (B.1.1.7)	Beta (B.1.351)	Delta (B.1.617.2)	Gamma (P.1)	Omicron(BA.1, BA.1.1)
**Abbott**	Anti-N IgG	100% (5/5)	100%(1/1)	100%(3/3)	100% (3/3)	100%(3/3)
**Abbott Quant**	Anti-S RBD IgG	100% (5/5)	0%(0/1)	100%(3/3)	100% (2/2)†	na*
**Ortho**	Anti-S IgG	100% (5/5)	0%(0/1)	100%(3/3)	100% (3/3)	100%(3/3)
**MSD**	Anti-N IgG	100% (5/5)	100% (1/1)	100%(3/3)	100% (3/3)	33%(1/3)
	Anti-S IgG	100% (5/5)	100% (1/1)	100%(3/3)	100% (3/3)	100%(3/3)
	Anti-S1 RBD IgG	100% (5/5)	0%(0/1)	100%(3/3)	100% (3/3)	100%(3/3)
	Combined	100% (5/5)	100% (1/1)	100%(3/3)	100% (3/3)	100%(3/3)
**ZEKMED**	Anti-S RBD IgM	80%(4/5)	100% (1/1)	100%(3/3)	100% (3/3)	0%(0/1)*
	Anti-S RBD IgG	80%(4/5)	0%(0/1)	67%(2/3)	67%(2/3)	100%(1/1)*
	Combined	100%(5/5)	100%(1/1)	100%(3/3)	100%(3/3)	100%(1/1)*
**PRNT_50_**	Anti-SARS-CoV-2 (Pango lineage B.1.2)	100% (5/5)	0%(0/1)	100%(3/3)	67%(2/3)	100%(3/3)

* Insufficient kits to perform assay.

† Only two specimens were tested due to insufficient sample volume.

### LOD and cross-reactivity

The WHO standard was used to estimate the LOD across assays for a direct, standardized comparison. LOD among all assays was within 1 log of each other, with Abbott Quant anti-S RBD having the lowest LOD of 9.9 BAU ml^−1^, followed by MSD assays (13.0 to 18.4 BAU ml^−1^), Ortho anti-S (16.9 BAU ml^−1^), Abbott anti-N (59.6 BAU ml^−1^) and PRNT_50_ (62.0 BAU ml^−1^) (Table 5). No cross-reactivity was detected among the six specimens tested for cross-reactivity.

**Table 5.** Limit of detection

Assay	Detection	LOD (BAU ml^−1^)
Abbott	Anti-N IgG	59.6
Abbott Quant	Anti-S RBD IgG	9.9
Ortho	Anti-S IgG	16.9
MSD	Anti-N IgG	13.0
	Anti-S IgG	18.4
	Anti-S1 RBD IgG	14.5
PRNT_50_	Anti-SARS-CoV-2 (Pango lineage B.1.2)	62.0

### Precision

All assays had 100% concordance in qualitative results for precision – positive or negative for detectable antibodies according to the assay’s cut-off. For the positive samples, Abbott, Abbott Quant, Ortho and MSD produced results with %CV<10% for both intra- and inter-assay precision (Table 6). PRNT_50_ results were within one (twofold) dilution, which is the acceptable range. Due to limited quantities of the assay kit, only intra-assay repeatability was performed for the ZEKMED POCT rapid diagnostic test and results had 100% concordance.

**Table 6.** Precision of positive samples

Assay	Detection	Intra-assay %CV or as described	Inter-assay %CV or as described
Abbott	Anti-N IgG	1.5%	2.1%
Abbott Quant	Anti-S RBD IgG	1.4%	4.2%
Ortho	Anti-S IgG	5.8%	7.5%
MSD	Anti-N IgG	0.8%	8.4%
	Anti-S IgG	0.5%	7.8%
	Anti-S1 RBD IgG	0.6%	6.8%
ZEKMED	Combined	All replicates were positive	na*
PRNT_50_	Anti-SARS-CoV-2 (Pango lineage B.1.2)	All replicates were within +/−1 dilution	All replicates were within +/−1 dilution

* Insufficient kits to perform assay.

## Discussion

We performed a comprehensive COVID-19 serology study with diverse types of qualitative and quantitative COVID-19 serology assays, including CMIA, EIA, multiplex EIA, rapid POCT and a PRNT assay that specifically detects neutralizing antibodies. Most of the studies in the literature that we know of focus on accuracy and cross-reactivity. Our study included LOD with a WHO standard and diagnostic characteristics on different variants. As the different assays have different cut-off values and read-outs, using a standardized WHO standard for determining LOD allowed a direct comparison. All medium-to-high throughput assays (Abbott, Ortho, MSD), POCT (ZEKMED) and PRNT assays evaluated in our study provided good PPA of ≥85% and high NPA of ≥90% compared to the reference panel. The CMIAs and EIAs had higher PPA (85.0–100%) than the POCT (43.3–85.0%). These results are consistent with other studies evaluating COVID-19 serology assays using patient convalescent sera [20,22,30].

In our study, assays that detected anti-S and anti-RBD antibodies provided higher PPA than those that detected anti-N antibodies. On both MSD and Abbott platforms, anti-S and anti-RBD IgG had higher PPA than anti-N IgG. As we did not know the time of collection of specimens post-infection, the lower PPA of the anti-N assays could be explained by the faster decline of anti-N antibodies over time relative to anti-S antibodies [23,36, 37]. When combining the detection of IgG to all targets in the MSD assay, PPA increased to 100%, and NPA decreased. Overall, the quantitative assays had false positive results; however, these values were close to the cut-off of the respective assay. It is also important to note that PRNT specifically detects neutralizing antibodies, whereas these assays detect neutralizing and binding antibodies. The quantitative assays also had lower LODs; therefore, it is possible that these ‘false positives’ reflect the detection of binding antibodies.

Although ZEKMED IgM alone had a lower PPA (43.3–53.3%), the combined IgM and IgG PPA was much higher (91.7–93.3%). The false negative specimens were due to the higher LOD of the POCT, as these specimens had values that were close to the cut-off using the Abbott Quant and MSD assay (data not shown). Since convalescent specimens were used, we expected to observe a poor PPA for IgM as it declines faster than IgG following seroconversion. These findings further reinforce the limited utility of serological testing in diagnosing acute SARS-CoV-2 infections [33].

Despite the numerous mutations in spike and nucleocapsid proteins of the VOCs, most of the COVID-19 serology assays we evaluated were able to detect antibodies generated from infections with various variants. This is consistent with what was reported by the manufacturers based on *in silico* analysis and other studies [24,26]. The Beta specimen was not reactive by most of the anti-S/S1 RBD IgG assays but was detected using the anti-N assays. Although we cannot firmly conclude that mutations in the spike protein of Beta VOC may impact antibody detection by COVID-19 serology assays that use the spike as the target, our findings are consistent with Reincke *et al*., which described reduced ELISA positivity and neutralization of 40 serum samples from patients infected with the Beta VOC compared to an ancestral strain [38]. One Gamma VOC specimen was negative by PRNT but was positive by the EIAs, suggesting that it is likely that only binding antibodies were present in the sample or the level of neutralizing antibody was below the LOD of our PRNT. The interpretation of the Omicron specimens was complicated by the fact that the patients were vaccinated and may have had both infection- and vaccine-induced antibodies. The N antigens can distinguish antibodies generated from infection versus vaccination; however, S and S1 RBD antigens do not. All the assays with S or S1 RBD antigens were able to detect antibodies from vaccinated Omicron-infected patients. The fact that the anti-N IgG assays were also able to detect antibodies from these patients suggests that the S/S1 RBD assays are detecting antibodies generated from the Omicron infection, in addition to those generated from vaccination. Notably, Abbott anti-N IgG, MSD anti-S IgG, MSD IgG with all three targets combined and combined ZEKMED anti-S RBD IgM/IgG assays were able to detect antibodies from all variants being studied.

In countries such as Canada where only spike-based vaccines are used, anti-N and anti-S detection has been successfully used to compare seroprevalence between infection-acquired and infection- or vaccine-acquired antibodies [39]. This has demonstrated the impact of vaccines and successive Omicron waves on seroprevalence in Canada. In addition, studies have evaluated the use of anti-N to detect infections in vaccinated and unvaccinated individuals, as well as combining the different targets of nucleocapsid, spike, RBD and antibody isotypes IgG, IgM and IgA to improve the prediction of time since infection [40,41]. Globally, other institutions have used serology to estimate the prevalence of infection in communities with varying vaccination rates and in vulnerable groups (e.g. older age, immunocompromised individuals), which can help to inform vaccination strategies [42,44]. One of the limited clinical uses of COVID-19 serology is in the diagnosis of multisystem inflammatory syndrome in children, a post-infectious inflammatory syndrome that can occur weeks after SARS-CoV-2 infection. Detection of SARS-CoV-2 antibodies provides laboratory evidence in individuals who have not been tested or are tested outside the window for PCR or antigen positivity [45]. In a vaccinated patient or where vaccination status is unknown, using an anti-N assay can help determine if the antibody is from infection with SARS-CoV-2.

SARS-CoV-2 neutralizing antibodies are thought to be primarily generated against the RBD region of the spike protein [46]. Our laboratory developed a PRNT assay that was based on the NML’s protocol [27,29]. The PPA was lower compared to the spike-based commercial EIAs. This is likely due to the lower LOD of the spike-based EIAs (9.9–18.4 BAU ml^−1^) compared to the PRNT_50_ (62.0 BAU ml^−1^). Neutralization was detected in 87% (13/15) of VOC sera specimens with our PRNT assay, which uses the SARS-CoV-2 strain with the pangolin lineage B.1.2 (close to ancestral SARS-CoV-2). This suggests that antibodies from VOC infection have some neutralization activity against ancestral SARS-CoV-2 and potentially provide cross-protection across different variants. Although recent studies have provided new insights on anti-S and anti-RBD titres and neutralizing antibodies as potential markers of protection against SARS-CoV-2 infection, a threshold or absolute correlate of protection has yet to be established [47,52]. Therefore, COVID-19 serology testing should not be used to define immunity to SARS-CoV-2.

Limitations of this study include lack of clinical information for the reference panel, in particular the time of collection from PCR positivity or symptom onset; small sample size for VOCs and cross-reactivity studies; limited sample from patients with other respiratory infections; and limited volumes or tests to perform all types of testing for every specimen (Table 4). The parainfluenza specimen was not tested with Abbott and Ortho due to insufficient sample volume and assay kits.

Overall, the MSD anti-S IgG assay had the best performance with 100% PPA and NPA. It was able to detect antibodies from patients infected with all variants included (Alpha, Beta, Delta, Gamma and Omicron) and had one of the lower LODs. However, the choice of COVID-19 serology assay will depend on the need or study objective. ZEKMED has the portability of being a POCT without the need for any equipment, and results are available in 20 min. This can be useful in remote areas where transport of samples can be challenging. The high-throughput MSD assay that simultaneously detects antibodies against spike, S1 RBD and nucleocapsid may be useful for serosurveillance studies where it is important to differentiate between infection-acquired and vaccination-acquired antibodies. The PRNT_50_ assay provides data on the detection of neutralizing or functional antibodies. This study evaluated different assays and provides metrics and limitations which may help in this decision-making.

## Conclusions

The assays in our study were designed using antigens from the ancestral strain of SARS-CoV-2. Our evaluation of VOC sera showed that these tests also have the ability to detect multiple variants. They are likely to detect antibodies from infection of emerging lineages and updates in vaccine strains going forward, which is important to vaccine- and infection-based serosurveillance studies [53,54]. The choice of a laboratory’s COVID-19 serology assay should depend on the specific context it is to be used in, and our findings can help inform this decision. Of the assays evaluated, the MSD anti-S IgG assay had the best performance in accuracy and the ability to detect all variants. It is likely that having assays to detect both anti-spike and anti-nucleocapsid antibodies would be practical, as it can support the identification of more recent exposures [39].

The varying units of measure and threshold cut-offs from the different assays highlight the need for standardization. In our study, the WHO international standard was used to determine LOD; further utilization can help standardize and compare quantitative results across assays [55,56]. COVID-19 serology plays an important role as a serosurveillance tool, contributing to our understanding of the transmission of SARS-CoV-2, serology studies with vaccine updates of new lineages, and the impact of public health measures throughout the pandemic. If a correlate of protection is discovered, the utility of COVID-19 serology may expand to determining immunity.

## Abbreviations

AU: arbitrary unit
BAU: binding antibody unit
CBS: Canadian Blood Services
CLIA: chemiluminescent immunoassay
CMIA: chemiluminescent microparticle immunoassay
CMV: cytomegalovirus
COVID-19: coronavirus disease 2019
%CV: coefficient of variation
DMEM: dulbecco's modified eagle medium
EIA: enzyme immunoassay
ELISA: enzyme-linked immunosorbent assay
HBV: hepatitis B virus
HCV: hepatitis C virus
HIV: human immunodeficiency virus
LOD: limit of detection
MMR: measles virus, mumps virus and rubella virus
MSD: Meso Scale Diagnostics
NML: National Microbiology Laboratory
NPA: negative percent agreement
PFU: plaque-forming unit
POCT: point-of-care test
PPA: positive percent agreement
PRNT: plaque reduction neutralization test
PSO: post-symptom onset
RBD: receptor binding domain
SARS-CoV-2: severe acute respiratory syndrome coronavirus 2
S/Co: signal-to-cut-off ratio
VOCs: variants of concern
